# Assessing Ultrasonography as a Diagnostic Tool for Porcine Cysticercosis

**DOI:** 10.1371/journal.pntd.0005282

**Published:** 2017-01-05

**Authors:** Robert H. Flecker, Ian W. Pray, Saul J. Santivaňez, Viterbo Ayvar, Ricardo Gamboa, Claudio Muro, Luz Maria Moyano, Victor Benavides, Hector H. Garcia, Seth E. O’Neal

**Affiliations:** 1 School of Public Health, Oregon Health & Science University and Portland State University, Portland, Oregon, United States of America; 2 School of Sciences, Department of Microbiology, Universidad Peruana Cayetano Heredia, Lima, Peru; 3 Instituto Peruano de Parasitologia Clinica y Experimental - INPPACE, Lima, Peru; 4 Center for Global Health Tumbes, Universidad Peruana Cayetano Heredia, Tumbes, Peru; Yeshiva University Albert Einstein College of Medicine, UNITED STATES

## Abstract

**Background:**

*Taenia solium* inflicts substantial neurologic disease and economic losses on rural communities in many developing nations. “Ring-strategy” is a control intervention that targets treatment of humans and pigs among clusters of households (rings) that surround pigs heavily infected with cysticerci. These pigs are typically identified by examining the animal’s tongue for cysts. However, as prevalence decreases in intervened communities, more sensitive methods may be needed to identify these animals and to maintain control pressure. The purpose of this study was to evaluate ultrasonography as an alternative method to detect pigs heavily infected with *T*. *solium* cysts.

**Methodology/Principal Findings:**

We purchased 152 pigs representing all seropositive animals villagers were willing to sell from eight communities (pop. 2085) in Piura, Peru, where *T*. *solium* is endemic. Tongue and ultrasound examinations of the fore and hind-limbs were performed in these animals, followed by necropsy with fine dissection as gold standard to determine cyst burden. We compared the sensitivity and specificity of ultrasonography with tongue examination for their ability to detect heavy infection (≥ 100 viable cysts) in pigs. Compared to tongue examination, ultrasonography was more sensitive (100% vs. 91%) but less specific (90% vs. 98%), although these differences were not statistically significant. The greater sensitivity of ultrasound resulted in detection of one additional heavily infected pig compared to tongue examination (11/11 vs. 10/11), but resulted in more false positives (14/141 vs. 3/141) due to poor specificity.

**Conclusions/Significance:**

Ultrasonography was highly sensitive in detecting heavily infected pigs and may identify more rings for screening or treatment compared to tongue examination. However, the high false positive rate using ultrasound would result in substantial unnecessary treatment. If specificity can be improved with greater operator experience, ultrasonography may benefit ring interventions where control efforts have stalled due to inadequate sensitivity of tongue examination.

## Author Summary

*Taenia solium* is a cestode that infects humans and pigs. The parasite causes up to one-third of epilepsy in Latin America, Asia and Africa and results in economic harm to smallholder farmers who cannot sell the contaminated pork of their infected pigs. “Ring-strategy” is an intervention being evaluated as a potential method to control the spread of infection within communities. This strategy involves identifying heavily infected pigs and targeting treatment resources to humans and pigs living nearby these animals. Tongue examination of pigs is used to provide a simple yet crude technique for identifying the most heavily infected pigs. The purpose of this study was to evaluate the ability of ultrasonography to identify *T*. *solium* infection in pigs and to compare it to traditional tongue examination methods. We found that ultrasonography may be better at detecting heavily infected pigs than traditional tongue examination methods, but has limitations such as increased cost and a high false positive rate. With improvements in training and greater operator experience, ultrasound may have the potential to contribute to control interventions based on ring-strategy.

## Introduction

Ultrasonography is a noninvasive diagnostic tool that can detect soft-tissue larval cestode infections. It is considered a primary method for identifying hepatic cystic hydatidosis and subcutaneous cysticercosis in humans [1–5], and has proved useful for mass screening of hydatidosis in sheep and goats [6–8]. However, an overlooked application for ultrasound is its potential use in the detection of cysticercosis in live pigs.

Cysticercosis occurs in pigs infected with larval cysts of the pork tapeworm, *Taenia solium*. Humans are the definitive hosts of the adult-stage parasite (taeniasis), which infects the small intestine and sheds eggs or gravid proglottids into the host’s feces. *T*. *solium* eggs are deposited into the environment through the stool of infected humans and later consumed by foraging pigs. Once ingested, the eggs develop into their larval form (cysticercosis), which encyst in the pig’s muscle or other soft tissue. The parasite’s life cycle is completed when a larval cyst in raw or undercooked pork is consumed by a human, and subsequently develops into an adult tapeworm in the small intestine.

A set of reliable diagnostic tools for porcine cysticercosis is needed to allow treatment of infected pigs prior to slaughter, to identify transmission hotspots within communities, and to monitor progress of control programs [9,10]. Only one study has evaluated the use of ultrasonography for detecting porcine cysticercosis [11]. While this study confirmed the ability of ultrasound to diagnose larval cysts in live pigs, only a small number of heavily infected animals were examined. Given that the vast majority of infected pigs have only a few cysts in the entire body [12–14], the utility of ultrasonography as an adequate screening tool for food safety purposes or for monitoring progress of control programs is doubtful. However, ultrasonography could prove useful for applications in which detection of heavily infected pigs is the goal. One such application is a control intervention known as “ring-strategy”, which involves screening and treatment of humans and pigs for taeniasis and cysticercosis, respectively, if they live within 100 meters of a pig heavily infected with cysticerci [15, 16]. The working assumption of this approach is that pigs with hundreds or thousands of infecting cysts have experienced repeated or intense exposure to *T*. *solium* eggs, suggesting that a human with taeniasis resides nearby, and that other humans and pigs in the area may be at increased risk of infection. This approach was developed in response to a small study in rural Peru that found the prevalence of taeniasis to be eight times greater among humans living within 100 meters of heavily infected pigs [17].

Studies of ring-strategy have relied on tongue examination of pigs to diagnose heavy cyst infection [16,18]. This method involves visual inspection and palpation of the inferior surface of the tongue in live pigs, and was traditionally performed by local buyers experienced at screening pigs prior to sale at market. Despite the appeal of tongue examination as a low-cost and locally accepted method, it suffers from a lack of sensitivity, as some studies have found that tongue examination fails to detect cysts in 80% or more of infected pigs [19,20]. There is evidence, however, to suggest that tongue examination performs better when used to exclusively identify heavily infected pigs. In a small Zambian study, researchers found that 35% of pigs with 100 or more cysts (compared to 0% of pigs with <100 cysts) were positively identified through tongue examination [19]. While more research is needed to confirm this hypothesis, it is likely that the sensitivity of tongue examination increases at even heavier cyst burdens, which can range into the thousands of cysts [12–14]. This makes it a reasonable tool for ring-strategy, which aims to identify only the most heavily infected pigs in a community.

Nonetheless, a rapid diagnostic test that can be applied in the field and that improves upon the sensitivity of tongue examination is needed in later stages of control. During early stages of control interventions, when the prevalence of porcine cysticercosis is high in endemic communities, even low sensitivity methods will identify heavily infected pigs and result in screening and treatment being applied.

As prevalence of both porcine cysticercosis and taeniasis decline, however, more sensitive methods are needed to identify and treat remaining pockets of infection. The purpose of this study, therefore, was to evaluate ultrasonography as an alternative to tongue examination for non-invasive detection of viable *T*. *solium* cysts in live pigs. We aimed to compare the performance of these two diagnostic tools for detecting pigs with heavy cyst burdens in the context of ring-strategy.

## Methods

### Study Site and Selection of Subjects

Our study consisted of eight villages in Piura (total population 2,085 residents), a province in the arid northern region of Peru where *T*. *solium* is endemic. We performed a door-to-door survey of all households in the eight villages and attempted to capture all pigs older than two months of age, with age verified by the owner. Eligible pigs were manually restrained while trained study personnel performed tongue examination and collected blood samples from each pig.

### Serologic Assessment

We collected five milliliters of blood from pigs in the field using pre-caval venipuncture, and stored samples in chilled ice coolers until they could be centrifuged in a field laboratory. 1 ml aliquots of sera were placed in microtubules, frozen at -20°C, and then shipped by air to the Cysticercosis Unit at the National Institute of Neurological Sciences in Lima for further analysis. Pig sera were analyzed by enzyme-linked immunoelectrotransfer blot (EITB) for presence of antibodies against *T*. *solium* cysts using methods described elsewhere [21]. Briefly, the EITB assay uses an enriched fraction of homogenized *T*. *solium* cysts containing seven *T*. *solium* glycoprotein antigens, GP50, GP42, GP24, GP21, GP18, GP14, GP13. Reaction to any of these seven glycoprotein antigens is considered positive. Of the 827 pigs tested from the eight study villages, 432 (52%) seropositive pigs were identified. Field teams returned to these households and offered to purchase all seropositive pigs in order to perform ultrasound examination and necropsy off-site. Due to the reluctance of villagers to sell their animals, only 152 (35%) seropositive pigs were purchased and underwent further testing at the Center for Global Health in Tumbes, Peru. We purchased seropositive pigs only in order to increase the likelihood of including pigs with viable cyst infection in the subsequent evaluations using necropsy and ultrasonography.

### Tongue Examination

Tongue examination was performed in the study community while the pig was manually restrained. We used a wooden stick to keep the mouth open while retracting the tongue with a cloth and visually inspecting and palpating the entire inferior aspect of the tongue for the presence of viable cysts. Pigs were considered tongue-positive if one or more fluid-filled cystic structures was either seen or felt, regardless of whether a central opacity was visible. Degenerated or calcified cysts, which can be confused with scars or granulomas resulting from tongue trauma, were excluded.

### Ultrasound Examination

**Fig 1:**
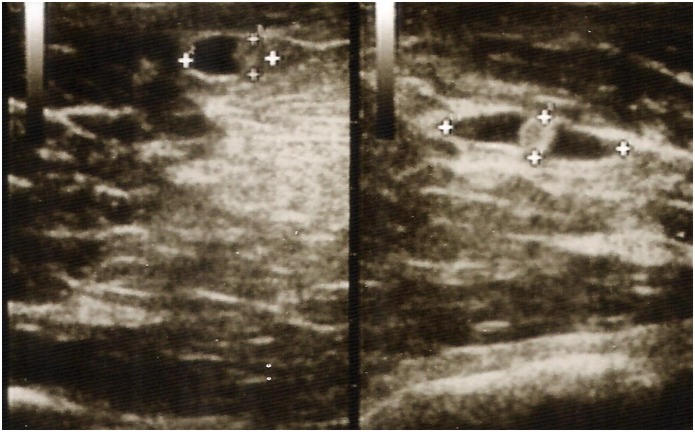
Viable *Taenia solium* cysticerci detected by ultrasonography of a live pig using a 5.0–10.0 MHz linear transducer. The cystic structures have clearly delineated borders, clear vesicular fluid, and a central opacity corresponding to the protoscolex.

Ultrasound examinations were conducted in the corrals at the Center for Global Health by a trained ultrasonographer who had experience using ultrasound to screen for intra-abdominal hydatid disease among humans and ruminants. Pigs were manually restrained on the ground in dorsal recumbency by two technicians securing the front and hind-legs while the medial aspects of both thighs and brachii were inspected for viable cysts using a SonoSite plus portable machine with a L38 5.0–10.0 MHz transducer (SonoSite, Bothell, WA, USA). For ultrasound examination, viable cysts were defined as cystic structures with clearly delineated borders containing clear vesicular fluid and a central opacity (Fig 1). The total number of viable cysts was recorded regardless of whether degenerating or calcified cysts were also encountered. We limited our analysis to viable cysts only as these presumably indicate more recent infection than do degenerated or calcified cysts, an important consideration in the context of ring-screening in which the goal is to identify and treat the active source of infection. To emulate field conditions, ultrasound examinations were restricted to a total of five minutes per animal. Following ultrasound examination, 0.1 mg/kg of xylazine combined with 5 mg/kg of ketamine was administered intravenously to provide a deep plane of anesthesia. The animal was then humanely euthanatized by injecting an overdose of sodium pentobarbital (100 mg/kg) intravenously.

### Necroscopic Examination

Detailed necropsy was conducted by systematically dissecting the full carcass of all pigs, and identifying any cysts present in the brain, heart, tongue and all skeletal muscles. Fine cuts of less than 0.5 centimeters were used to dissect all inspected tissues and the total number of viable cysts in each pig was recorded. Viable cysts were defined as well-delineated thin-walled cystic structures containing clear vesicular fluid and a visible white protoscolex. The total number of viable cysts encountered was recorded. Degenerated and calcified cysts were not considered in this analysis. For pigs with particularly dense cyst burdens, a weighed sample of forelimb muscle was counted for cysts and extrapolated to estimate the total body burden.

### Statistical Analysis

Data analysis was performed using Stata version 14.0 (Stata Corp., College Station, TX, USA). Necropsy cyst burden was stratified as negative (no cysts), light for 1–9 viable cysts, moderate for 10–99 viable cysts, and heavy for those with more than 100 viable cysts. We then compared tongue examination with ultrasound detection for their ability to detect heavily infected pigs (≥100 viable cyst identified on necropsy). In an initial analysis, we found that visualizing just one viable cyst on ultrasound was responsible for 46% (12/26) of false positive pigs. Therefore, to improve specificity, we required the identification of at least two viable cysts on ultrasound to meet the definition of positive for subsequent analyses. We compared the sensitivity and specificity of the two screening methods and calculated exact binomial 95% confidence intervals for each. We also calculated and plotted positive and negative predictive values of each test under a range of hypothetical prevalences of heavily infected pigs to assess performance of each under a variety of endemic scenarios.

### Ethics

This study was reviewed and approved by the Institutional Review Boards and Institutional Ethics Committees for the Use of Animals at Oregon Health & Science University, Portland, Oregon, USA under permit number IS00002843, and the Universidad Peruana Cayetano Heredia, Lima, Peru under permit 61326. Treatment of animals adhered to the Council for International Organizations of Medical Sciences (CIOMS) International Guiding Principles for Biomedical Research Involving Animals.

## Results

### Swine Characteristics and Serology

The median age of the 152 study pigs was 10 months (range: 6 to 32 months). Nearly 80% (120/152) were under 1 year of age and 55% (84/152) were female. Weight ranged between 7 and 74 kilograms (mean: 28 kg). Serologic results revealed that 40% (60/152) of pigs had one or two reactive bands on EITB, 34% (51/152) had three reactive bands, and 27% (41/152) had four or more reactive bands.

### Necroscopic Examination

Among the 152 pigs necropsied, 105 (69%) did not have any viable cysts, 27 (18%) had a light cyst burden (1 to 9 cysts), 9 (6%) pigs had a moderate burden (10 to 99 cysts), and 11 (7%) had a heavy burden (≥100 cysts) (Table 1). Among the 11 heavily infected pigs, the cyst burden ranged from 545 to over 34 thousand cysts (median: 2,827 cysts); 10 (91%) had over 1000 cysts.

**Table 1:** Number of viable cysts identified on necropsy compared to tongue and ultrasound examination.

Necropsy burden (viable cysts)	No. (%)	No. (%) with positive tongue examination	No. (%) positive on ultrasonography
0	105(69.1)	2 (1.9)	11 (10.5)
1–9	27 (17.8)	0 (0)	1 (3.7)
10–99	9 (5.9)	1 (11.1)	2 (22.2)
100–999	1 (0.7)	0 (0)	1 (100.0)
≥1000	10 (6.6)	10 (100)	10 (100)

### Tongue Examination

Inspection of the inferior aspect of the tongue identified cysts in 8.6% of pigs (13/152). 10 out of 11 heavily infected pigs (≥100 viable cysts by necropsy) were positively identified, yielding a sensitivity for detecting heavy infection of 90.9% (95% CI: 58.7, 99.8) (Table 2). The pig with heavy infection that was not detected by tongue examination had the lowest cyst burden among heavily infected pigs (545 viable cysts on necropsy). Tongue examination positively identified 10 out of 10 pigs that had at least 1000 cysts. Tongue examination was positive in 2/105 (1.9%) pigs that were negative on necropsy and 1/36 (2.8%) pigs with light to moderate cyst burden. Combined, these positive tongue findings in lightly infected or uninfected pigs (3/141) resulted in a specificity of 97.9% (95% CI: 93.9, 99.6) for detecting heavy cyst burden in pigs (≥100 viable cysts).

**Table 2:** Sensitivity and specificity of tongue examination and ultrasonography for detecting pigs with different burdens of viable cysticerci.

	Tongue Examination		Ultrasonography	
Necropsy burden (viable cysts)	Sensitivity % (95% CI)	Specificity % (95% CI)	Sensitivity % (95% CI)	Specificity % (95% CI)
≥1	23.4 (12.3, 38.0)	98.1 (93.3, 99.8)	29.8 (17.3, 44.9)	89.5 (82.0, 94.7)
≥10	55.0 (31.5, 76.9)	98.5 (94.6, 99.8)	65.0 (40.8, 84.6)	90.9 (84.7, 95.2)
≥100	90.9 (58.7, 99.8)	97.9 (93.9, 99.6)	100.0 (71.5, 100)	90.1 (83.9, 94.5)
≥1000	100.0 (69.2, 100)	97.9 (94.0, 99.6)	100.0 (69.2, 100)	89.4 (83.2, 94.0)

### Ultrasound Examination

**Fig 2:**
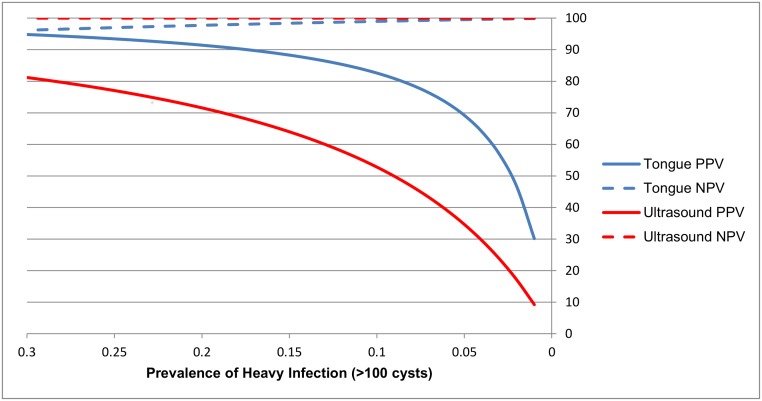
Positive and negative predicted values for the detection of heavily infected pigs (≥ 100 cysts) with ultrasonography and tongue examination projected across a range of prevalence values.

We required a minimum of two viable cysts to be identified by ultrasound to consider the result to be positive. At least two suspected cysts were seen in 16% (25/152) of pigs. 11 out of 11 heavily infected pigs (≥100 viable cysts by necropsy) were positively identified with ultrasonography, yielding a sensitivity for detecting heavy infection of 100% (95% CI: 71.5, 100.0) (Table 2). Ultrasound was positive in 11/105 (10.5%) pigs that were negative by necropsy and 3/36 (8.3%) pigs with light to moderate cyst burdens. Taken together, these positive ultrasound findings in lightly infected or uninfected pigs (14/141) yielded a specificity of 90.1% (95% CI: 83.9, 94.5) for detecting heavy cyst burden (≥ 100 viable cysts). The positive (PPV) and negative predictive values (NPV) for tongue examination and ultrasonography across a range of prevalence values for heavily infected pigs are shown in Fig 2. While the NPV was similar for both methods at the low prevalences (<10%) typically seen in endemic areas in Peru, the PPV of tongue examination was substantially higher than that for ultrasound at all prevalence levels.

## Discussion

The main purpose of this study was to compare ultrasonography to tongue examination as a rapid diagnostic tool for identifying live pigs heavily infected with viable *T*. *solium* cysts, and to determine the utility of ultrasonography in the context of a ring-strategy. Although the modest improvement in sensitivity of ultrasonography, as compared to tongue examination, could potentially increase control pressure through the identification of more heavily infected pigs and associated areas of parasite transmission, the lower specificity would translate into application of substantial treatment resources in areas that may not have increased risk. Unless the specificity of ultrasonography can be improved, development of alternative methods for identifying heavy infection in pigs, such as rapid serology, should be pursued. In addition, the results of this study demonstrate that neither ultrasonography nor tongue examination accurately identify pigs with low or moderate infection burdens. This confirms that neither are adequate diagnostic tools for use in food safety monitoring or for measuring progress of control programs.

The 9% difference in sensitivity between ultrasonography and tongue examination (100% vs. 91%) was not statistically significant, and in absolute terms translated to just one additional heavily infected pig being detected among the 152 pigs analyzed. Nonetheless, if the greater sensitivity of ultrasound represents a true difference, it could have an impact on ring interventions given the high reproductive capacity of the adult stage tapeworm and the potential for an untreated case to reverse control gains. In a recent investigation that allocated treatment rings based on tongue-positivity in pigs, the prevalence of taeniasis at study end was 1.4% in the treatment arm [16]. While this was significantly less than the prevalence in the control arm (2.5%), the majority of remaining taeniasis carriers went untreated because they did not fall into treatment rings, suggesting a potential underdiagnosis of heavily infected pigs in this trial. To advance ring control efforts beyond what is possible when using tongue examination, a more sensitive method of diagnosing heavily infected pigs is needed.

Ultrasound may also allow for the detection of pigs with slightly lower cyst burdens than tongue examination, which may be beneficial in later stages of control when there are fewer infected pigs present at any level of cyst burden. While both ultrasound and tongue examination performed extremely well in identifying pigs with massive cyst burdens (100% sensitivity for pigs with ≥1000 cysts), only ultrasound identified the one pig that was found to have 545 cysts. This may simply reflect that ultrasound allowed us to screen a greater mass of skeletal muscle than did tongue examination. The ability of ultrasound to detect infected pigs with lower thresholds of cyst burden could prompt screening and treatment intervention in areas that may otherwise have been missed.

Despite ultrasound’s potential greater sensitivity compared to tongue examination, the high false positive rate we observed precludes its use in ring-screening unless specificity can be improved. Even after we required a minimum of two cysts to be detected on ultrasound to meet the definition of positive, ultrasonography had lower specificity for detecting pigs with heavy cyst burdens compared to tongue examination (90% vs. 98%), leading to an unacceptably low positive predictive value at all but the highest background prevalences for heavily infected pigs. Our concern is that by using ultrasonography as a diagnostic tool in the context of a ring strategy, unnecessary treatment rings would be created, leading to substantial over-treatment of humans and pigs as compared to tongue examination.

Although we found that ultrasonography critically lacked specificity in this study, it is an operator-dependent test and performance will likely improve with experience. The ultrasound examinations in this study were performed by a single operator, who, although trained and experienced in the detection of inter-abdominal hydatid cysts in small ruminants, had no prior experience diagnosing cysticerci in pigs. With more experience, the ability to discriminate cysts from other structures may improve. Conversely, it is unlikely the performance of tongue examination is subject to significant improvement. Since ultrasonography and tongue examination have poor sensitivity at lower cyst burdens, neither is an adequate screening tool for evaluating food safety or for monitoring progress of control programs. Results from several necropsy studies in endemic villages have shown that the vast majority of pigs infected with cysticercosis have less than 10 cysts in the entire carcass [16–18]. Neither ultrasonography nor tongue examination was able to accurately diagnose pigs with burdens of infection less than 100 cysts, detecting just 8% (3/36) and 3% (1/36) of these infected pigs respectively. Serologic methods or carcass dissection, despite known limitations, continue to be the predominant methods used to monitor the effectiveness of control interventions [10,22], while there is no reliable tool available for food safety applications. The development of a rapid serologic test that could detect heavily infected pigs with high specificity (>98%) and moderately high sensitivity (>90%), could provide a viable option for use in ring strategy.

Evaluating ultrasonography and tongue examination requires important qualitative comparisons. Ultrasonography incurs additional costs including the equipment and the service of a skilled technician. Although the higher cost could be justified in the later stages of control when greater sensitivity is needed, ultrasonography provides little value above tongue examination in the early stages of a ring intervention. However, ultrasonography does provide a potential benefit of creating an opportunity for education and engagement with community members intrigued by the ability to visualize cysts in their pigs on the ultrasound screen. This level of engagement is beyond that which we observed during tongue examination or serum collection, and may ultimately improve trust between community members and project staff, and increase community knowledge about cysticercosis prevention.

We chose to include only seropositive pigs (based on EITB assay) in our sample in order to maximize the number of heavily infected pigs examined. While EITB measures antibody response, and not necessarily active cyst infection, antigen detection assays are known to cross-react with *T*. *hydatigena* which is highly prevalent in the study region. It is important to note that although the exclusion of seronegative pigs deliberately biased our sample towards including a greater proportion of pigs with viable cysts, this does not affect the sensitivity and specificity values that we report for ultrasonography or tongue examination. However, the PPV and NPV are strongly influenced by the underlying prevalence, which is why we chose to present these characteristics across a range of prevalence values. Future studies would benefit from a larger sample of pigs to increase the precision of point estimates for sensitivity and specificity in each stratum of cyst burden. While it is possible that pig age, sex and weight might influence the performance of ultrasound due to the amount of fat present in the animal, our sample included pigs across a broad range of the characteristics. Lastly, we chose the medial aspects of the fore and hind limbs for ultrasonography as these sites are readily accessible, are sparsely haired, and are known to harbor cysts. Other anatomic sites, however, may allow for clearer visualization of cysts or may have higher predilection for cyst formation, ultimately affecting the sensitivity and specificity of the test.

## Supporting Information

S1 Appendix Supplemental Table. Sensitivity and specificity of ultrasonography (any viable cyst present) for detecting pigs with different burdens of viable cysticerci.
(DOCX)

S2 Appendix Study dataset. Characteristics, serology, necropsy, tongue examination, and ultrasound examination data of 152 study pigs.
(XLS)
