# Supplementary material for: Assessing Ultrasonography as a Diagnostic Tool for Porcine Cysticercosis
Source: PLoS Negl Trop Dis. 2017 Jan 5;11(1):e0005282. doi: 10.1371/journal.pntd.0005282 (PMC5242540; doi:10.1371/journal.pntd.0005282)
Supplement: S1 Appendix — Sensitivity and specificity of ultrasonography (any viable cyst present) for detecting pigs with different burdens of viable cysticerci. (DOCX) [file pntd.0005282.s001.docx]

Supplemental table. Sensitivity and specificity of ultrasonography (any viable cyst present) for detecting pigs with different burdens of viable cysticerci (n=152 pigs).

|  | Ultrasonography (≥1 cyst) | |
| --- | --- | --- |
| Necropsy burden  (viable cysts) | Sensitivity  % (95% CI) | Specificity  % (95% CI) |
| ≥1 | 42.6 (28.3. 57.8) | 83.8 (75.3, 90.3) |
| ≥10 | 80.0 (56.3, 94.3) | 84.1 (76.7, 89.9) |
| ≥100 | 100.0 (71.5, 100) | 81.6 (74.2, 87.6) |
| ≥1000 | 100.0 (69.2, 100) | 81.0 (73.6, 87.1) |
